# The role of mycorrhizal associations in plant potassium nutrition

**DOI:** 10.3389/fpls.2014.00337

**Published:** 2014-07-17

**Authors:** Kevin Garcia, Sabine D. Zimmermann

**Affiliations:** Biochimie et Physiologie Moléculaire des Plantes, UMR 5004 CNRS/INRA/SupAgro/UM2Montpellier, France

**Keywords:** potassium, plant nutrition, arbuscular mycorrhizal symbiosis, ectomycorrhizal symbiosis, transport systems

## Abstract

Potassium (K^+^) is one of the most abundant elements of soil composition but it's very low availability limits plant growth and productivity of ecosystems. Because this cation participates in many biological processes, its constitutive uptake from soil solution is crucial for the plant cell machinery. Thus, the understanding of strategies responsible of K^+^ nutrition is a major issue in plant science. Mycorrhizal associations occurring between roots and hyphae of underground fungi improve hydro-mineral nutrition of the majority of terrestrial plants. The contribution of this mutualistic symbiosis to the enhancement of plant K^+^ nutrition is not well understood and poorly studied so far. This mini-review examines the current knowledge about the impact of both arbuscular mycorrhizal and ectomycorrhizal symbioses on the transfer of K^+^ from the soil to the plants. A model summarizing plant and fungal transport systems identified and hypothetically involved in K^+^ transport is proposed. In addition, some data related to benefits for plants provided by the improvement of K^+^ nutrition thanks to mycorrhizal symbioses are presented.

## Introduction

Potassium (K^+^) is one of the most important macronutrient for all organisms. In plants, K^+^ represents 2–10% of the dry biomass, its optimal cytoplasmic concentration for enzyme activities being around 100–200 mM (Leigh and Wyn Jones, 1984). This cation participates to various crucial processes such as plasma membrane polarization, growth, stomatal aperture, or adaptation to environmental changes (Broadley and White, 2005; Wang and Wu, 2013; Anschütz et al., 2014; Shabala and Pottosin, 2014). Maintaining an elevated K^+^ concentration in plant cells is vital for the smooth running of such physiological processes (Benito et al., 2014; Shin and Adams, 2014). Although K^+^ ions are extremely abundant in soil, their availability is very low due to their strong mineral adsorption. Depending on soil type, the K^+^ concentration in soil solution is approximately 0.1–1 mM (Asher and Ozanne, 1967). This low availability combined to the constitutive demand of plants lead to the formation of depletion areas around roots (Drew and Nye, 1969). Consequently, plants need to develop efficient strategies to improve the K^+^ uptake from soil (Nieves-Cordones et al., 2014; Zörb et al., 2014), such as the acquisition of high-affinity transport systems or the establishment of plant-microbe associations.

Mycorrhizal symbioses are mutualistic interactions between the root systems of around 80% of land plants and the mycelium of various fungi (Wang and Qiu, 2006). Among mycorrhizal associations, two forms are mainly studied due to their ecological importance, arbuscular mycorrhizae (AM) and ectomycorrhizae (ECM). Mycorrhizal fungi participate actively to plant development (Smith and Read, 2008) by improvement of access to nutrients, particularly when resources become scarce. In turn, vegetal partners provide up to 20–25% of photosynthetic carbohydrates to their symbionts (López et al., 2008). The improvement of plant nutrition through mycorrhizal symbioses and the molecular bases of nutrient transfer are currently well studied for phosphorus (Javot et al., 2007; Plassard and Dell, 2010) and nitrogen (Müller et al., 2007; Jin et al., 2012). However, only few data concern the possible mycorrhizal contribution to K^+^ acquisition (Benito and Gonzalez-Guerrero, 2014).

Herewith, we compiled and summarized the current knowledge concerning the role of AM and ECM symbioses in plant K^+^ nutrition. An overview of transport systems acting putatively in transfer of K^+^ from soil to fungal cells and from fungi to plant cells is highlighted. Finally, improvement of K^+^ acquisition by mycorrhizal associations will lead to benefits for the plant in diverse environmental conditions.

## Evidence of plant potassium nutrition by mycorrhizal symbiosis

### Arbuscular mycorrhizal symbiosis

Plant K^+^ nutrition through the arbuscular mycorrhizal pathway has been rarely studied. However, assessment of potassium distribution in AM fungi (*Rhizophagus irregularis*) using particle-induced X-ray emission (PIXE) experiments (Johansson and Campbell, 1988) revealed a strong K^+^ accumulation in spores (Pallon et al., 2007), hyphae (Olsson et al., 2008), and vesicles (Olsson et al., 2011). Interestingly, PIXE analyses showed a higher K^+^ concentration in root-sections of *Aster tripolium* mycorrhized by *R. irregularis* than non-inoculated plants, suggesting a possible increase of K^+^ acquisition due to the AM colonization (Scheloske et al., 2004). Such a K^+^ enrichment of plants mycorrhized by various AM fungi was also observed in *Zea mays* root steles (Kaldorf et al., 1999), in *Pelargonium peltatum* shoots (Perner et al., 2007) and in *Lactuca sativa* leaves (Baslam et al., 2013). K^+^ transport was commonly visualized by the utilization of rubidium (Rb^+^) as an analog tracer. Measuring AM fungi mediated Rb^+^ uptake from the soil, Hawkes and Casper (2002) showed putative competition mechanisms for four herbaceous species.

### Ectomycorrhizal symbiosis

Potassium fluxes from ECM fungi to host plants were first observed by determination of Rb^+^ contents (Rygiewicz and Bledsoe, 1984; Jongbloed et al., 1991). Quantification of K^+^ in *Picea abies* cultivated in a medium with 230 μM of K^+^ resulted in around 5–6% of total K^+^ that came from the ECM fungus *Paxillus involutus* (Jentschke et al., 2001). Regarding the cellular distribution of K^+^ within fungal hyphae, X-ray microanalysis showed localization mainly in vacuoles of the ECM fungus *Pisolithus tinctorius* (Orlovich and Ashford, 1993; Ashford et al., 1999). PIXE experiments on *P. sylvestris* / *Suillus luteus* mycorrhizae revealed a high K^+^ concentration in ectomycorrhizae vascular tissues (Turnau et al., 2001). Excitingly, data obtained in multiple *Rhizopogon* sp. isolates from field showed an important K^+^ sequestration in rhizomorphs, that could be vital for forests subjected to long periods of K^+^ deprivation (Wallander et al., 2002; Wallander and Pallon, 2005). Another fungus that can be considered as an important K^+^ accumulator is *Suillus granulatus* (Wallander et al., 2003). Strong mineral degradation capacities of these two latter ECM fungi were suggested by the identification of calcium-rich crystals originating from K^+^-rich mineral apatite on rhizomorph surfaces. Thus, *Rhizopogon* sp. and *S. granulatus* could be considered as key facilitators between soil and trees for K^+^ fluxes in temperate forest ecosystems. Recently, an increase of K^+^ contents of about 35% was observed in *Pinus pinaster* mycorrhized by *Hebeloma cylindrosporum* upon 2 months culturing in K^+^ deficiency, suggesting that this fungus plays a crucial role in pine adaptation to limiting conditions (Garcia et al., 2014). K^+^ assimilation was improved also in shoots of *Acacia spirorbis* and *Eucalyptus globules* mycorrhized by *Pisolithus albus* up to 38% (Jourand et al., 2014). By contrast, *Quercus ilex* and *Quercus faginea* colonized by *Tuber melanosporum* displayed a significant reduction of K^+^ concentrations (Dominguez Nunez et al., 2006). However, in another experiment, no difference in K^+^ contents was observed between control plants of *Pinus halepensis, Q. faginea*, and *Quercus petraea*, and those inoculated with *T. melanosporum* (Dominguez Nunez et al., 2008). These contradictory data highlighted once again that K^+^ allocation from soil to plants through ECM fungi need complementary functional investigations.

## Transport of potassium in mycorrhizal interactions

### Transport systems on the fungal side

**Figure 1:**
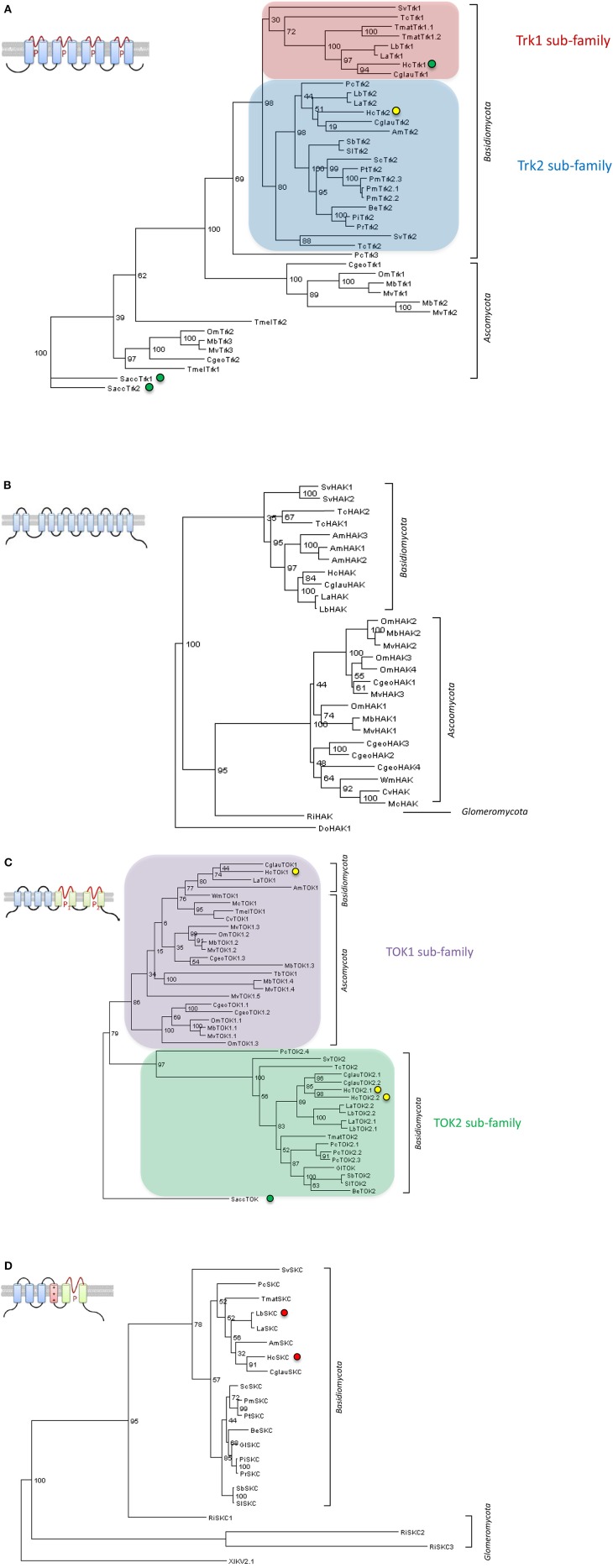
**Phylogenetic trees of potassium transport systems from sequenced mycorrhizal fungi**. Four families of K^+^ transport systems were identified in sequenced mycorrhizal fungi. Two of them code for putative transporters, for the **(A)** Trk and **(B)** HAK families, and the two others for putative ion channels, for the **(C)** TOK and **(D)** Shaker-like (SKC) families, respectively. **(A)** Two sub-families of Trk transporters were identified in *Basidiomycota*. **(C)** Two subfamilies of TOK channels were found in *Asco-* and *Basidiomycota*. Neither of these two families were identified in the *Glomeromycota* species *Rhizophagus irregularis*. **(D)** No SKC channel was found in *Ascomycota* fungi (mycorrhizal or not), suggesting the loss of this channel in this clade. Structure models of each family are represented. Transmembrane domains are symbolized by rectangles and pore domains by a P. The voltage-dependent domain of SKC proteins **(D)** is indicated by a red rectangle (+++). Trees were constructed using maximum likelihood method with 100 bootstraps. Green, yellow and red circles indicate successful published, successful unpublished (Zimmermann et al., unpublished data) or failed unpublished functional characterization, respectively. Am, *Amanita muscaria*; Be, *Boletus eludis*; Cgeo, *Cenococcum geophilum*; Cglau, *Cortinarius glaucopus*; Cv, *Choiromyces venosus*; Hc, *Hebeloma cylindrosporum*; Gl, *Gyrodon lividus*; La, *Laccaria amethystina*; Lb, *Laccaria bicolor*; Mb, *Meliniomyces bicolor*; Mc, *Morchella conica*; Mv, *Meliniomyces variabilis*; Om, *Oidiodendron maius*; Pc, *Piloderma croceum*; Pi, *Paxillus involutus*; Pm, *Pisolithus microcarpus*; Pr, *Paxillus rubicundulus*; Pt, *Pisolithus tinctorius*; Ri, *Rhizophagus irregularis*; Sb, Suillus brevipes; Sc, *Scleroderma citrinum*; Sl, Suillus luteus; Sv, *Sebacina vermifera*; Tb, *Terfezia boudieri*; Tc, *Tulasnella calospora*; Tmat, *Tricholoma matsutake*; Tmel, *Tuber melanosporum*; Wm, *Wilcoxina mikolae*. Lengths of aligned sequences are around 1000, 800, 900, and 500 amino acids for Trk, KUP, TOK, and SKC proteins, respectively. *Saccharomyces cerevisiae* [SaccTrk1 and SaccTrk2 **(A)**, SaccTOK **(C)**], *Debaryomyces occidentalis* [DoHAK1 **(B)** and *Xenopus laevis* XlKV2.1 **(D)**] protein sequences were selected as outgroups to root trees. All sequences were picked up on the mycorrhizal fungi JGI genome portal: http://genome.jgi.doe.gov/Mycorrhizal_fungi/Mycorrhizal_fungi.info.html.

Recent access to genomes of ECM fungi *Laccaria bicolor* (Martin et al., 2008), *T. melanosporum* (Martin et al., 2010) and many others (http://genome.jgi.doe.gov/Mycorrhizal_fungi/Mycorrhizal_fungi.info.html) allows the identification of new candidate genes involved in mycorrhizal resource exchanges (Casieri et al., 2013). Consequently, four families of putative K^+^ transport systems could be identified in ECM fungi (Figure 1) on the basis of their homology to yeast Trk transporters (Ko and Gaber, 1991), to yeast TOK channels (Ketchum et al., 1995), to bacterial and yeast KT/KUP/HAK transporters (Bossemeyer et al., 1989; Bañuelos et al., 1995) and to animal *Shaker*-like channels (SKC) (Papazian et al., 1987; Jan and Jan, 1997). Before availability of these genomes, a Trk-type transporter and a *Shaker*-like channel were identified in an EST library of *H. cylindrosporum* (Lambilliotte et al., 2004). The member of the Trk/Ktr/HKT family (Corratgé-Faillie et al., 2010) was functionally characterized. *Hc*Trk1 was shown to restore partially the wild-type phenotype of a yeast strain deficient in K^+^ uptake (Corratgé et al., 2007). Moreover, electrophysiological analyses performed by expression of cRNA in *Xenopus* oocytes argued that *Hc*Trk1 might function as a Na^+^-K^+^ transporter. More recently, the use of *H. cylindrosporum* transgenic lines allowed the localization of this transporter exclusively in external hyphae of *P. pinaster* mycorrhizae (Garcia et al., 2014), suggesting a specialized reorganization of *Hc*Trk1 to uptake sites. The other candidate identified in the *H. cylindrosporum* EST library belongs to the SKC family representing voltage-dependent K^+^-selective channels (Lambilliotte et al., 2004). Interestingly, SKC channels were found exclusively in *Basidiomycota* fungi and in some members of basal fungi, whereas they are absent in sequenced *Ascomycota*, suggesting a loss of these K^+^ channels in this clade. Excitingly, the *H. cylindrosporum* genome accession provides now two additional types of transport systems, three *Hc*TOK channels and a *Hc*HAK transporter. The analysis of these new candidates is currently in progress in order to dissect the whole K^+^ transportome of *H. cylindrosporum*.

Transporters and channels of AM fungi are still the missing part of the K^+^ transport from soil to host. Four sequences of *R. irregularis* from an EST library (http://mycor.nancy.inra.fr/IMGC/GlomusGenome/index3.html) were identified as K^+^ transport systems (Casieri et al., 2013). Three of them are coding for SKC-type channels and one for a KT/KUP/HAK transporter. Interestingly, no Trk and TOK members were identified from this library, and even from the sequenced nuclear genome (http://genome.jgi.doe.gov/Gloin1/Gloin1.home.html). Functional characterization and analysis of these new candidates will provide more precise information on fungal molecular players involved in AM plant K^+^ nutrition.

**Figure 2:**
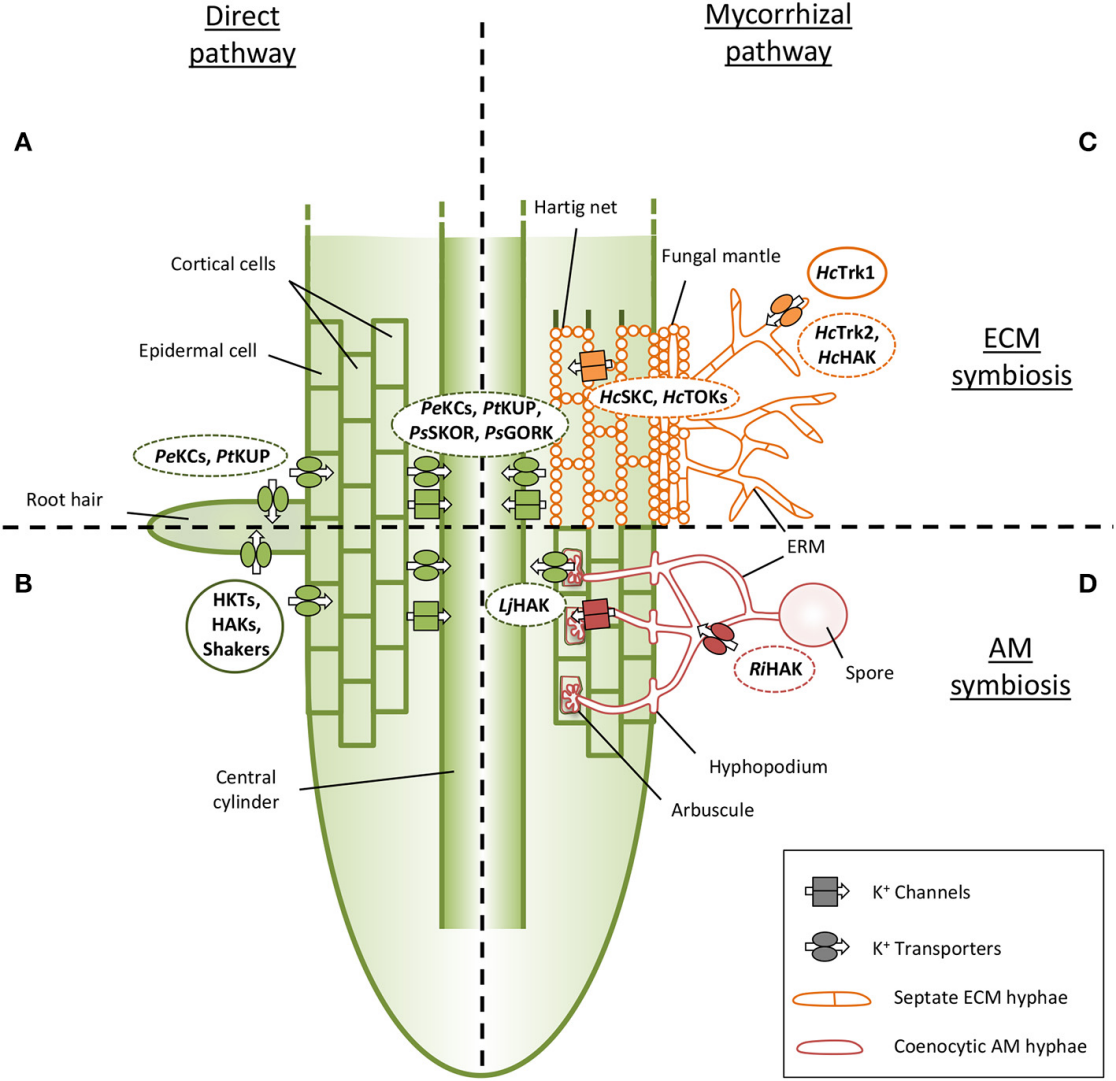
**Current knowledge about potassium transport systems in mycorrhizal associations. (A,B)** The transfer of potassium (K^+^) from the soil to plant cells by the direct pathway needs plant transport systems in root uptake and release sites. **(A)** Shaker type channels from *Populus euphratica* (*Pe*KCs) and a KUP transporter of *Populus trichocarpa* (*Pt*KUP) could be involved in K^+^ uptake in poplar trees (Zhang et al., 2010). **(B)** Members of HKT, HAK and Shaker families were identified and characterized in various AM plants as transport systems involved in K nutrition. **(C)** To our knowledge, only K^+^ transport systems of the fungus *Hebeloma cylindrosporum* are currently analyzed in ECM symbiosis, and just one, *Hc*Trk1, is already characterized (Corratgé et al., 2007, Garcia et al., 2014). Few transcriptomic data suggest the role of some plant proteins allowing K^+^ uptake from the apoplasm. **(D)** EST library of *Rhizophagus irregularis* allows the identification of several K^+^ transport related proteins (Casieri et al., 2013), and transcriptomic analysis revealed an high overexpression of a K^+^ transporter in inoculated *Lotus japonicus* (Guether et al., 2009). Full lines indicate transport systems whose capability in K^+^ transport was verified. Dotted lines indicate transport systems whose involvement in K^+^ transport during mycorrhizal symbioses is suggested. ECM fungal structures: Extraradical hyphae (ERM), fungal mantle and Hartig net. AM fungal structures: ERM, spore, hyphopodium and arbuscule. Plant root cells: roots hair, epidermal cells and cortical cells, central cylinder. *Hc, Hebeloma cylindrosporum; Ri, Rhizophagus irregularis; Pe, Populus euphratica; Pt, Populus tremula; Ps, Pinus sylvestris; Lj, Lotus japonicus*.

Future research on AM and ECM K^+^ transport systems need to precise their putative involvement in K^+^ uptake from the soil or in K^+^ release toward plant cells. Based on homology, it is tempting to argue that transporters of the Trk/Ktr/HKT and KT/KUP/HAK families could take up K^+^ from the soil. Similarly, *Shaker*-like K^+^ and TOK channels could be probably involved in the transfer of K^+^ from the arbuscule or Hartig net to host plant cortical cells (Figure 2; Benito and Gonzalez-Guerrero, 2014). However, caution must be taken on these predictions due to the possible bidirectional behavior of some transport systems in specific conditions and due to their unknown subcellular localization.

### Transport systems on the plant side

The direct pathway of woody plant K^+^ uptake (Figure 2A) is poorly studied so far in contrast to model plants as, e.g., *Arabidopsis thaliana* (Alemán et al., 2011; Dreyer and Uozumi, 2011; Coskun et al., 2014; Nieves-Cordones et al., 2014). However, some K^+^ transport systems in woody plants able to form ECM associations have been identified. For example, two channels expressed in roots of *Populus euphratica* (*Pe*KC1 and *Pe*KC2) were characterized (Zhang et al., 2010). Interestingly, their over-expression led to complementation of the *A. thaliana akt1* mutant. Because AKT1 is involved in K^+^ acquisition in *A. thaliana* (Hirsch et al., 1998), this finding strongly suggests that these two transport systems could play a role in K^+^ nutrition in poplar trees. However, their role in context of mycorrhizal associations has not been dissected. Their expression analysis upon AM and ECM symbioses would be a good starting point for the identification of transport systems required in K^+^ nutrition, as already shown for phosphate transporters of *Populus trichocarpa* (Loth-Pereda et al., 2011). In turn, in AM plants, the direct pathway of K^+^ uptake is well known since many years (Figure 2B), several transport systems were identified, functionally characterized and their role in K^+^ nutrition in various conditions was investigated. These proteins belong to the transporter families Trk/Ktr/HKT (Corratgé-Faillie et al., 2010) and KT/KUP/HAK (Grabov, 2007), as well to the *Shaker*-like channels (Dreyer and Uozumi, 2011).

Very few studies investigated so far the plant K^+^ transportome of the mycorrhizal pathway (Figures 2C,D). Recently two ESTs of *P. sylvestris* representing the SKOR-type outward *Shaker*-like channel were found to be highly up-regulated during ECM interaction with *Ceonococcum geophilum*, and less up-regulated upon interaction with *S. granulatus* and *Rhizopogon roseolus* (Martina Peter, pers. comm.). A KT/KUP/HAK transporter was found to be 44-fold up-regulated in *Lotus japonicus* mycorrhized by the AM fungus *Gigaspora margarita* (Guether et al., 2009). More recently, a SKOR channel of *Z. mays* was identified to be up-regulated by AM colonization in response to salinity (Estrada et al., 2013). Future analyses of these first plant candidates are needed to dissect the molecular bases of K^+^ uptake from the plant-fungus interface.

## Benefits of mycorrhizal potassium uptake for plants

### Improvement of salt and drought stress tolerance

Advantages conferred by K^+^ originating from mycorrhizae include improved stress tolerance of the host plant. Acquisition of plant salinity tolerance by AM symbiosis has been described for several decades (Hirrel and Gerdemann, 1980; Ojala et al., 1983), very little is known about physiological and molecular mechanisms enhancing this adaptation. AM colonization enhances the plant K^+^ uptake whereas the Na^+^ content is maintained at low levels in salt stress conditions applied to *Vicia faba* (Rabie and Almadini, 2005), *Acacia nilotica* (Giri et al., 2007), *Ocimum basilicum* (Zuccarini and Okurowska, 2008), *Glycine max* (Sharifi et al., 2007), *Olea europaea* (Porras-Soriano et al., 2009), or *Z. mays* (Estrada et al., 2013). These data indicate that AM symbiosis improves salt tolerance of the host plant through the modification of the K^+^/Na^+^ balance. This finding was corroborated by high internal K^+^ concentrations in several AM fungi collected in natural saline sites (Hammer et al., 2011a). Recently, Estrada et al. (2013) demonstrated differential expression levels during AM colonization for three K^+^ transport systems of *Z. mays* putatively involved in phloem loading/unloading (*Zm*AKT2), xylem release (*Zm*SKOR), and Na^+^/K^+^ homeostasis (*Zm*SOS1). These exciting findings open the way to the elucidation of plant proteins involved in transport of K^+^ originating from mycorrhizal fungi, especially under salt stress conditions.

Input of AM symbiosis on drought stress resistance of plants has been well studied (Harley and Smith, 1983; Al-Karaki, 1998; Porcel and Ruiz-Lozano, 2004). Improvement in hydric stress tolerance is accompanied by an elevation of K^+^ concentrations observed, e.g., in *Citrus tangerine* (Wu and Xia, 2006), indicating a role of AM symbiosis through K^+^ uptake required for osmotic adjustment. Interestingly, El-Mesbahi et al. (2012) demonstrated that hydraulic conductivity of AM-colonized *Z. mays* growing under hydric stress was enhanced by supply of K^+^ in external medium. Moreover, the expression level of the plant aquaporin *Zm*PIP2;6 was modulated by K^+^ supply in hydric stress, suggesting a tight link between adaptation of mycorrhized plants to drought stress and K^+^ resource availability.

Salt and drought stress tolerance linked to potassium nutrition has been so far less studied in ECM plants. However, recently, Danielsen and Polle (in press) have been shown an increase of K^+^ in ECM poplar under drought conditions suggesting also in this symbiotic interaction a role of mycorrhizal K^+^ for environmental stress adaptation.

### Interaction between plant potassium and phosphorus nutrition

Interestingly, a strong correlation between K^+^ and phosphorus (P) during AM symbiosis was reported. Olsson et al. (2008), (2011) highlighted a co-distribution and a linked ratio of K^+^ and P in *R. irregularis* spores, hyphae and vesicles. When spores were enriched in P, an increase of K^+^ content was observed (Olsson et al., 2011). Several studies on ECM symbiosis reported similar results. Indeed, a strong correlation in K^+^ and P distribution was described in rhizomorphs of *Rhizopogon* sp. using PIXE analyses (Wallander and Pallon, 2005). Other studies showed that the decrease of P availability in soil could lead to either a decrease or an increase of K^+^ content in ECM with *Pinus rigida* (Cumming, 1993) or in AM with *Trifolium subterraneum* (Smith et al., 1981), respectively. Moreover, K^+^ and P were found in same fungal compartments of *P. involutus* such as vacuoles (Orlovich and Ashford, 1993; Ashford et al., 1999). Interestingly, it is assumed that K^+^ is one of the major counter-ions of polyphosphate (polyP) granules, especially of soluble polyP short-chains mainly located in fungal vacuoles (Bücking and Heyser, 1999). Recently, elemental analysis of spherical electron-opaque granules in the vacuoles of *Scleroderma verrucosum* hyphae associated with *Quercus acutissima* using TEM-EDS (Transmission electron microscopy-energy-dispersive Xray Spectroscopy) showed major correlated peaks for P and K (Jung and Tamai, 2013). In addition, we have revealed recently that the over-expression of a K^+^ transporter of *H. cylindrosporum* led to an alteration of K^+^ and P translocation from roots to shoots of mycorrhized *P. pinaster* under K^+^ deprivation (Garcia et al., 2014), providing new evidences for K^+^ and P interaction during their transport in ECM symbiosis. All these data demonstrating the strong link between these two elements suggest that K^+^ seems to be a more important component of mycorrhizal symbiosis than formerly suspected. Therefore, K^+^ needs to be considered not only as a direct trophic element involved in plant K^+^ nutrition, but also as an “indirect-trophic” component required for homeostasis and correct transfer of other nutrients to the host plant, such as inorganic phosphate. Moreover, Hammer et al. (2011b) described a K^+^ accumulation in an AM fungus related to low C supply from the plant. Consequently, we can imagine that in conditions of low availability of C originating from the host plant, the high concentration of K^+^ observed in the fungus could be related to the accumulation of P (in polyP form) which is not transferred to the plant. However, additional investigations are needed to validate or not this hypothesis and to get more insight on the interaction occurring between K^+^, P and C in mycorrhizal symbioses.

### Protection against radiocaesium pollution

Radiocaesium isotopes (^134^Cs, ^137^Cs) are important soil contaminants that can enter the food chain by the intermediate of plant uptake (Delvaux et al., 2001). It is well known that external K^+^ affects the acquisition of radiocaesium by plants (Tamponnet et al., 2008). Involvement of mycorrhizal symbiosis on radiocaesium uptake was reported. Evaluation of the Cs^+^/K^+^ ratio in *P. abies* showed a lower acquisition of ^134^Cs by plants inoculated with *Hebeloma crustuliniforme* due to its retention in the outer hyphae and to a better transfer of K^+^ to the plant (Brunner et al., 1996). By contrast, *P. pinaster* mycorrhized by *R. roseolus* displayed more elevated concentrations in ^134^Cs than non-mycorrhized plants, whereas the K^+^ content remained stable (Ladeyn et al., 2008), highlighting the importance of the considered host-symbiont couple. The influence of K^+^ on ^134^Cs accumulation was also investigated in AM symbiosis, in *Medicago truncatula* colonized by *R. irregularis*. Radiocaesium accumulation of plants seems to be inversely correlated to K^+^ contents in the external medium (Gyuricza et al., 2010a). Based on these results, AM symbiosis combined with high K^+^ concentrations in external medium would be crucial to avoid ^134^Cs accumulation in plants growing on contaminated soil. Interestingly, external P elicits the same effects as K^+^ on ^134^Cs uptake during AM colonization of *M. truncatula* (Gyuricza et al., 2010b), reinforcing the idea of a close relationship between K^+^ and P via polyP synthesis, storage and transport. In contrast to these studies, pot experiments by Joner et al. (2004) with different external K^+^ concentrations, three host plants and two AM fungal species indicated that AM effects on plant ^134^Cs and ^137^Cs accumulation could be negligible. Further investigations will be needed to conclude whether mycorrhizal associations play a direct, via transport processes, or an indirect role on plant protection to radiocaesium contaminated soils.

## Conclusion

Although the role of K^+^ is still poorly investigated in mycorrhizal studies, it appears that plant K^+^ nutrition is clearly improved by mycorrhization, especially under K^+^ limiting conditions as, e.g., found in forest ecosystems. Moreover, this improvement could act on abiotic stress tolerance, P homeostasis maintenance, or exclusion of soil contaminants such as radiocaesium. Thanks to genome and transcriptome access, the dissection of molecular mechanisms involved will be unraveled in the coming years, strengthening our knowledge on the mycorrhizal contribution to plant K^+^ nutrition.

### Conflict of interest statement

The authors declare that the research was conducted in the absence of any commercial or financial relationships that could be construed as a potential conflict of interest.
